# Deep Learning-Driven Automatic Segmentation of Weeds and Crops in UAV Imagery †

**DOI:** 10.3390/s25216576

**Published:** 2025-10-25

**Authors:** Jianghan Tao, Qian Qiao, Jian Song, Shan Sun, Yijia Chen, Qingyang Wu, Yongying Liu, Feng Xue, Hao Wu, Fan Zhao

**Affiliations:** 1 Graduate School of Global Environmental Studies, Sophia University, Tokyo 102-8554, Japan; 2 Faculty of Robot Science and Engineering, Northeastern University, Shenyang 110819, China; 3 Graduate School of Frontier Sciences, The University of Tokyo, Tokyo 277-8563, Japan; 4 College of Information Science and Engineering, Xinjiang College of Science & Technology, Urumqi 830046, China; 5 Department of Environmental Health Sciences, University of California, Los Angeles, CA 90095, USA; 6 Graduate School of Information, Production and Systems, Waseda University, Tokyo 169-8050, Japan; 7 Graduate School of Information Science and Technology, The University of Tokyo, Tokyo 113-8654, Japan

**Keywords:** automatic segmentation, crops-weed detection, deep learning, precision agriculture, super-resolution reconstruction, UAV remote sensing

## Abstract

Accurate segmentation of crops and weeds is essential for enhancing crop yield, optimizing herbicide usage, and mitigating environmental impacts. Traditional weed management practices, such as manual weeding or broad-spectrum herbicide application, are labor-intensive, environmentally harmful, and economically inefficient. In response, this study introduces a novel precision agriculture framework integrating Unmanned Aerial Vehicle (UAV)-based remote sensing with advanced deep learning techniques, combining Super-Resolution Reconstruction (SRR) and semantic segmentation. This study is the first to integrate UAV-based SRR and semantic segmentation for tobacco fields, systematically evaluate recent Transformer and Mamba-based models alongside traditional CNNs, and release an annotated dataset that not only ensures reproducibility but also provides a resource for the research community to develop and benchmark future models. Initially, SRR enhanced the resolution of low-quality UAV imagery, significantly improving detailed feature extraction. Subsequently, to identify the optimal segmentation model for the proposed framework, semantic segmentation models incorporating CNN, Transformer, and Mamba architectures were used to differentiate crops from weeds. Among evaluated SRR methods, RCAN achieved the optimal reconstruction performance, reaching a Peak Signal-to-Noise Ratio (PSNR) of 24.98 dB and a Structural Similarity Index (SSIM) of 69.48%. In semantic segmentation, the ensemble model integrating Transformer (DPT with DINOv2) and Mamba-based architectures achieved the highest mean Intersection over Union (mIoU) of 90.75%, demonstrating superior robustness across diverse field conditions. Additionally, comprehensive experiments quantified the impact of magnification factors, Gaussian blur, and Gaussian noise, identifying an optimal magnification factor of 4×, proving that the method was robust to common environmental disturbances at optimal parameters. Overall, this research established an efficient, precise framework for crop cultivation management, offering valuable insights for precision agriculture and sustainable farming practices.

## 1. Introduction

*Nicotiana tabacum*, commonly known as tobacco, ranks among the most significant global cash crops, with its market value increasing from approximately $253 billion in 2022 to $266 billion in 2023, reflecting a compound annual growth rate (CAGR) of 5.4% [1]. China stood as the largest tobacco producer in 2021, followed by Brazil, India, and Indonesia, all of which have substantial tobacco cultivation areas. Within China, Yunnan Province contributed the largest share, over 50% of the country’s total tobacco production.

However, the expansion of tobacco farming has had profound implications for land use and environmental sustainability. The environmental repercussions include deforestation for firewood used in curing tobacco leaves, degradation of soil fertility, pollution of groundwater and surface water, and detrimental impacts on surrounding ecosystems, ultimately threatening national biodiversity [2,3]. Moreover, intensified weed competition has been closely linked to yield reductions [4,5]. Traditional weed management relies on manual weeding, which is labor-intensive and raises ethical concerns, while herbicide application offers an alternative but requires precise timing and dosage to be effective. Research indicates that inappropriate herbicide use can reduce crops productivity and increase environmental risks [6,7]. For example, yield losses up to 15% have been reported when weeds reach 10 inches in height in corn fields [8]; the inappropriate use of herbicide is related to soil and water contamination, biodiversity loss, and heavy metal accumulation [9,10,11]. These challenges highlight the urgent need for efficient and precise weed management strategies in tobacco cultivation.

The integration of precision agriculture into tobacco farming can be effectively achieved through precision sensing and image processing technologies. The principle of precision agriculture is to tailor cultivation practices to intra-field variability such as soil texture, topography, and vegetation cover, thereby enabling more efficient input management [12,13]. In recent years, deep learning-based image processing has become a powerful tool in this field, with applications covering crop classification, weed detection, and real-time farm management [14,15,16].

Existing studies can be broadly grouped into three research directions. The first focuses on remote sensing combined with convolutional neural networks (CNNs), which have demonstrated strong performance in classifying crops and weeds at field and UAV scales [17,18]. The second emphasizes real-time and robotic applications, where CNN models are embedded into autonomous platforms for tasks such as robotic weed detection and precision spraying [19]. A third line of work explores enhanced network architectures and data strategies, including residual CNNs with data augmentation, which improve accuracy and robustness under challenging agricultural conditions [20,21].

Despite these advances, tobacco cultivation presents unique challenges that are not well addressed by existing studies. First, the heterogeneity and complexity of tobacco field backgrounds, including soil, other vegetation types, and occasionally shadows, hinder the algorithms’ capacity for precise segmentation between tobacco plants and weeds [22,23]. On another front, the visual similarities in green hues and morphologies shared between weeds and tobacco plants, particularly at their nascent growth phases, result in frequent misclassifications and reduced segmentation precision for machine learning models [22,24]. Furthermore, variability induced by fluctuations in illumination, meteorological conditions, and growth stages substantially impacts the visual representation of both tobacco and weeds in imagery, complicating consistent recognition [23,25]. The resolution provided by remote sensing technologies may fall short of detailing necessities vital for distinguishing minor or closely situated weeds from tobacco plants, which is essential for ensuring precise herbicide application and avoiding damage to the crop. Consequently, it became imperative to innovate upon existing deep learning-based crop segmentation approaches to cater to the unique demands of tobacco cultivation scenarios [26,27].

Recent progress in deep learning–based Super-Resolution Reconstruction (SRR) offers a promising direction. SRR techniques are designed to restore high-resolution details from low-resolution images by learning complex nonlinear mappings. They have been widely validated in fields such as medical diagnostics [28,29] and autonomous vehicular technologies [30,31]. In the context of UAV imaging, SRR has proven particularly valuable, enhancing the visual fidelity of captured images and thereby improving the performance of subsequent computer vision tasks [32,33,34]. Among these tasks, semantic segmentation and object detection are among the most widely applied, benefiting significantly from SRR-enhanced imagery [26,35,36].

Building on these insights, this study aims to establish a UAV-based framework that integrates super-resolution reconstruction with semantic segmentation to improve crop-weed discrimination in tobacco fields. The framework is evaluated using both image quality metrics (PSNR, SSIM) for SRR models and segmentation accuracy metrics (IoU, mIoU) for semantic segmentation models. The contributions of this study can be summarized in three aspects. First, to the best of our knowledge, this is the first work that integrates UAV-based imagery, super-resolution reconstruction, and semantic segmentation for monitoring tobacco fields. While previous UAV- and SRR-related studies have primarily addressed crops such as soybean, wheat, maize, or blueberry [4,18,25], this study proposes a technical solution to address the distinctive visual challenges of tobacco cultivation, including fine venation patterns, strong visual similarity between weeds and tobacco plants, and heterogeneous soil backgrounds. Second, whereas most prior studies have relied on CNN-based segmentation models, this work systematically evaluates state-of-the-art Transformer models (DINOv2) and Mamba-based networks, and further develops an ensemble approach to enhance segmentation accuracy. This provides the first benchmark analysis of these newly proposed architectures compared to traditional CNN-based architectures in the context of tobacco monitoring. Third, the annotated dataset generated in this study is made publicly available, supporting reproducibility and facilitating future research in precision agriculture.

This article was structured as follows. Section 2 delineated the methodological framework, encompassing the data processing, SRR algorithms, segmentation models, and evaluation metrics. Section 3 describes dataset preparation, SRR experiments, and segmentation results. Section 4 discusses the influence of key parameters and compares improvement mechanisms. Section 5 drew a comprehensive conclusion of the whole article.

## 2. Methodology

**Figure 1:**
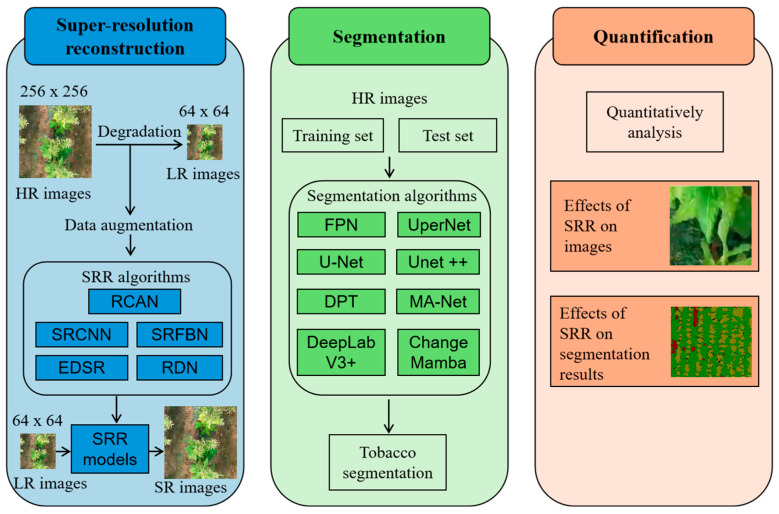
Flowchart of proposed framework.

Figure 1 illustrates the methodological framework and main procedures applied in this research. Initially, HR images of tobacco plantations, captured at 256 × 256 pixels using a consumer-grade UAV, were downsampled to 128 × 128 pixels to generate LR counterparts. A training set composed of these LR-HR pairs, along with selected SRR models, was employed to learn the complex nonlinear mapping between the two resolutions and subsequently to reconstruct SR mappings from the LR mappings. These SR outputs were then subjected to pixel-wise segmentation via a pre-trained model tailored for tobacco identification, enabling precise pixel-level classification. Finally, the effectiveness of different SRR techniques was evaluated by analyzing the segmentation outcomes, offering insights into the spatial distribution of tobacco plants across the field. Further procedural details are available in Section 2.4 and Section 2.5.

### 2.1. Survey Site

**Figure 2:**
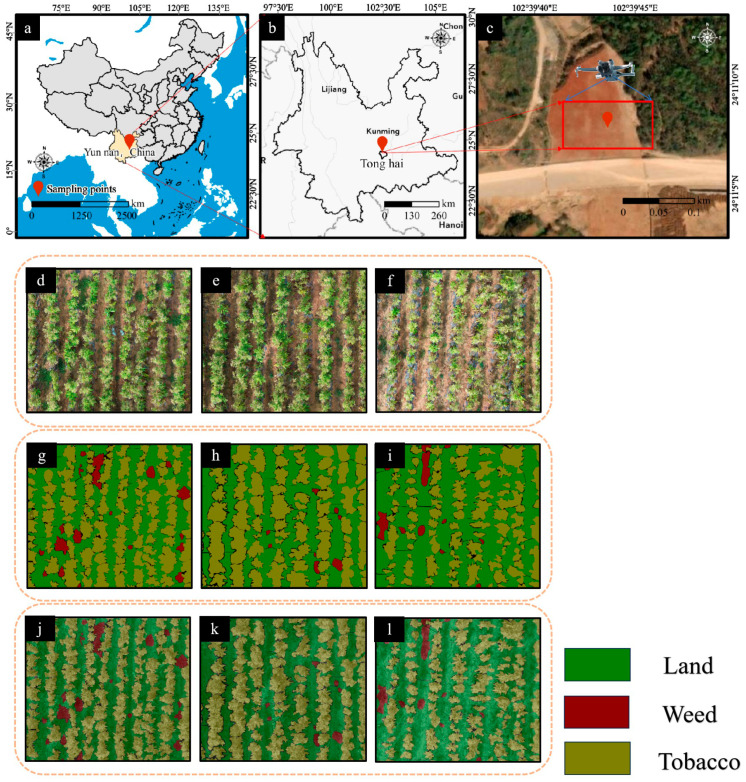
(**a**) The location of Yunnan Province delineated by Chinese administrative boundaries; (**b**) The location of Tonghai County within the city of Yuxi; (**c**) Key research areas of tobacco fields; (**d**–**l**) Visualizations of the labeled areas and mask annotations in partial tobacco field regions.

The chosen survey site for tobacco planation was situated in Yunnan Province, China, shown in Figure 2, which contributed 38.6% of the total tobacco yield in China and encompasses 32.4% of the nation’s cultivation area, making it the undisputed leader in both production and scale. This dominance was attributed to its unique agroecological conditions: the subtropical highland climate provides an annual average temperature of 15–20 °C, rainfall of 1000–1500 mm, and 2200–2500 annual sunshine hours, which align closely with the optimal requirements for tobacco growth. Furthermore, the region’s elevation gradients (1000–2500 m above sea level) and iron-rich red soils, which cover 67% of the province’s arable land, enhanced nutrient availability and leaf quality, yielding tobacco with superior combustibility and aroma profiles [37].

### 2.2. Data Collection

For data collection in this study, a DJI Mini 3 drone (SZ DJI Technology Co., Ltd.) was utilized. With a weight of 248 g, the drone supported up to 38 min of flight time, featured an 82.1° field of view (FOV), and could capture images at a resolution of 4000 × 3000 pixels. To maximize image quality, data acquisition was conducted under clear weather conditions with mild wind. The drone flew at an altitude of 3 m to obtain images with the highest possible resolution for use as HR data, while maintaining a safe distance to minimize wind disturbance that could cause leaf movement and motion blur. The full survey of the plantation took 30 min, which was determined by the battery capacity and the need for sufficient overlap between adjacent images to enable orthophoto map generation.

### 2.3. Image Preprocessing

In the preprocessing phase, the orthophoto map of the entire tobacco field captured by UAV was subdivided into approximately 840 image patches of 256 × 256 pixels (HR images) using a sliding window approach with a patch size of 256 pixels and a stride of 32 pixels. These HR images were subsequently downsampled to 64 × 64 pixels (LR counterparts) using a degradation model. HR-LR pairs were used for training SRR models, whereas HR images alone were employed for training segmentation models. To increase dataset diversity, data augmentation strategies such as rotations, horizontal and vertical flips, and scaling were applied in both SRR and segmentation training pipelines, balancing computational demands and model performance.

The HR images were further annotated for training the semantic segmentation models. Specifically, HR images were manually annotated into class “land”, “weed”, and “tobacco” as shown in Figure 2 using LabelMe tool. This process generated corresponding annotated images in .png format. To ensure annotation quality, two domain experts independently performed the labeling and resolved discrepancies through discussion. Additionally, local tobacco farmers were invited to review the annotations and provide contextual validation. The verified images were then used to train and test all semantic segmentation models in this study. The annotated dataset has been made publicly available, and the access link is provided in the Data Availability section.

### 2.4. Super-Resolution Reconstruction

Figure 1 presents the workflow of the deep convolution network-based SRR techniques for improving the visual quality of LR imagery. For training purposes, a specific SR dataset was generated by artificially reducing the resolution of HR images to create LR inputs, while the original HR images were retained as ground truth. Recognizing the diversity in architectural designs among SRR models for extracting features from LR data, five representative deep learning methods were selected: Super-Resolution Convolutional Neural Network (SRCNN), Super-Resolution Feedback Network (SRFBN), Enhanced Deep Super-Resolution Network (EDSR), Residual Dense Network (RDN), and Residual Channel Attention Network (RCAN). During training, LR inputs were passed through these SRR models, with a loss function quantifying the deviation between the reconstructed SR outputs and the ground-truth HR images to guide optimization. Model performance was assessed using PSNR and SSIM metrics on previously unseen LR images. Based on the comparative analysis, the most effective SRR model was chosen to generate SR inputs for the downstream semantic segmentation task.

#### 2.4.1. Architecture of SRR Networks

This study investigated seven deep learning-based SRR techniques. One of the earliest among them, SRCNN [38], proposed a three-layer convolutional network to model the nonlinear transformation from LR to HR images. Although structurally simple, SRCNN effectively captured intricate image patterns and significantly outperformed traditional handcrafted methods, laying the groundwork for more advanced SR architectures. Expanding on residual network concepts, EDSR [39] removed batch normalization layers and deepened the network using additional residual blocks, thereby achieving top-tier results on standard benchmarks. This architecture proved particularly effective at capturing high-frequency image details, emphasizing the need for architecture-specific design in SRR. RDN [40] further extended residual learning by integrating dense connections and Residual Dense Blocks (RDBs), allowing the model to exploit information across all network layers for improved feature extraction and aggregation. The features from RDBs were then fused with global context, enabling the model to utilize both localized and holistic information for image generation. RCAN [40] introduced a channel attention mechanism to dynamically adjust channel-wise feature representations, thereby enhancing critical high-frequency details necessary for precise reconstruction. With its attention-augmented residual design, RCAN consistently performed well across benchmarks and demonstrated the effectiveness of attention mechanisms in low-level vision enhancement tasks.

#### 2.4.2. Training of the Networks

The training of SRR models was designed to iteratively optimize network parameters by minimizing the discrepancy between generated SR images and their HR counterparts. This process enabled the model to recover intricate structural and textural details inherent to HR images while maintaining fidelity to the underlying data distribution. For CNN-based architectures (e.g., EDSR, RCAN), the training objective centers on minimizing the L1 loss, which quantifies the absolute pixel-wise difference between the reconstructed SR image *R*(*i*, *j*) and the original HR image *O*(*i*, *j*), as defined below:

(1) $$L_{1}\left(O,R\right)=\frac{1}{pq}\Sigma_{i=0}^{p-1}\Sigma_{j=0}^{q-1}\left\|O\left(i,j\right)-R\left(i,j\right)\right\|$$

where *p* × *q* denotes the image resolution of original HR image. In contrast, GAN-based models (e.g., SRGAN, ESRGAN) incorporated perceptual and adversarial losses to balance pixel accuracy and perceptual realism. The perceptual loss leveraged feature maps extracted from a pre-trained VGG network to measure semantic consistency between SR and HR images:

(2) $$L_{perceptual}=\sum_{i=1}^{N}\;∥\phi_{i}(I_{SR})-\phi_{i}(I_{HR}){∥}_{2}^{2}$$

where *ϕi* represented the feature map at layer *i*. The adversarial loss, fundamental to GAN training, encourages the generator *G* to produce images indistinguishable from real HR data. For the generator, this was defined as $L_{adv}=-log(D(G(z)))$, where *z* denotes the LR input, while the discriminator *D* was optimized using:

(3) $$L_{D}=-\left(log(D(x))+log(1-D(G(z)))\right)$$

with *x* representing real HR images. The Adam optimizer was adopted for parameter updates, with an initial learning rate of 5 × 10^−5^ and a decay strategy that reduced the rate every 50 epochs. Training was conducted over 300 epochs using a batch size of 16 and implemented in PyTorch.

#### 2.4.3. Evaluation Metrics

The performance of SRR models was evaluated using two full-reference metrics: PSNR and SSIM [41]. PSNR measured pixel-level accuracy by computing the logarithmic ratio of the maximum possible pixel intensity to the Mean Squared Error (MSE) between the reconstructed SR image and the original HR image:

(4) $$MSE=\frac{1}{pq}\Sigma_{i=0}^{p-1}\Sigma_{j=0}^{q-1}{\left[O\left(i,j\right)-R\left(i,j\right)\right]}^{2}$$

(5) $$PSNR=10\times {lg}_{10}\left(\frac{{MAX}_{I}^{2}}{MSE}\right)$$

where *R*(*i*, *j*) represented the SR image and *O*(*i*, *j*) denoted the HR image with *p* × *q* resolution. *MAX*_*I*_, conventionally set to 255, represented the maximum gray value within the image. A higher PSNR value indicated better reconstructing performance for images.

SSIM assessed perceptual quality by comparing luminance, contrast, and structural details between HR and SR, aligning with the human visual system:

(6) $$SSIM\left(O,R\right)=\frac{\left(2\mu_{O}\mu_{R}+C_{1}\right)\left(2\sigma_{OR}+C_{2}\right)}{\left(\mu_{O}^{2}+\mu_{R}^{2}+C_{1}\right)\left(\sigma_{O}^{2}+\sigma_{R}^{2}+C_{2}\right)}$$

where $\mu_{O}$ and $\mu_{R}$ denote the mean pixel intensities of images *O* and *R*, respectively. $\mu_{O}^{2}$ and $\mu_{R}^{2}$ represent the variances, and $\sigma_{OR}$ is the covariance between the two images. The constants $C_{1}$ and $C_{2}$ are introduced to avoid instability caused by near-zero denominators. SSIM values lie within the range [0, 1], where larger values reflect better structural similarity between the images.

### 2.5. Tobacco Segmentation

#### 2.5.1. Network Architecture

Semantic segmentation, a fundamental task and widely used technique in computer vision (CV), involves assigning predefined category labels to individual pixels within an image, enabling fine-grained scene understanding. Deep learning-based segmentation methods exceled in this task by training on large annotated datasets, where neural networks learned intricate mappings between low-level visual features (e.g., color, texture) and high-level semantic labels (e.g., “tobacco leaf,” “weed”). These models leveraged hierarchical feature extraction and contextual reasoning to interpret complex spatial relationships, achieving robust performance in diverse environmental conditions. In this study, eight state-of-the-art semantic segmentation architectures: Feature Pyramid Networks (FPN), U-Net, DeepLabV3+, U-Net++, MA-Net, Dense Prediction Transformer (DPT), ChangeMamba, and UperNet, were selected to detect tobacco leaf across plantation-scale imagery.

##### FPN

FPN, introduced by Lin et al. (2017) [42], enhanced multi-scale object detection by constructing hierarchical pyramid feature maps. Unlike earlier models relying solely on final-layer features, where low-level features provided spatial precision and high-level features offered richer semantics, FPN integrated these features through a top-down approach. This strategy improved the detection of smaller objects without significantly increasing computational complexity.

##### UperNet

UperNet combined concepts from FPN and the Pyramid Pooling Module (PPM) to facilitate unified perceptual parsing [43]. Its design aimed to recognize diverse visual concepts within images and perform joint reasoning tasks. UperNet uniquely predicted pixel-level texture labels from image-level annotations, improving its capability to interpret visual data effectively.

##### U-Net

U-Net, proposed by Ronneberger et al. (2015) [44], features a distinctive U-shaped convolutional neural network structure composed of an encoder–decoder pair connected by skip connections. The encoder performed down-sampling, while the decoder reconstructs spatial detail via up-sampling. Skip connections preserved spatial details, aiding accurate localization. Nonetheless, U-Net had limitations concerning optimal network depth selection and constrained in skip connection configurations.

##### Unet++

Unet++, an advancement over U-Net, resolved these limitations by integrating multi-depth networks and redesigned skip connections to aggregate multi-scale features [45]. These improvements enhanced network flexibility and computational efficiency, resulting in faster inference speeds.

##### DeepLabV3+

DeepLabV3+ utilized an encoder–decoder framework featuring atrous and atrous separable convolutions, expanding receptive fields for multi-scale feature extraction [46]. After feature fusion, channel adjustment occurred through 1 × 1 convolution. Despite its strengths, DeepLabV3+ encounters challenged related to computational efficiency, especially with larger image sizes.

##### MA-Net

MA-Net incorporates the Position Attention Block (PAB) and the Multi-Scale Fusion Attention Block (MFAB), which leverage self-attention mechanisms to extract spatial interdependencies and capture fine-grained pixel-level relationships across the entire image [42], as well as to handle channel-wise dependencies, respectively. The integration of these attention modules enables the model to effectively capture comprehensive contextual features, enhancing the distinction between tobacco leaves, weeds, and background regions.

##### DPT

Dense Prediction Transformer (DPT), built upon Transformer architectures, processes HR feature representations, ensuring a global receptive field at each processing stage [47]. Leveraging the Transformer’s ability to capture long-range dependencies, DPT exceled in tasks requiring detailed, dense image predictions.

##### ChangeMamba

ChangeMamba introduced three spatiotemporal state space modeling mechanisms, including series modeling, cross modeling, and parallel modeling to enhance segmentation accuracy [30]. Combined with the Mamba architecture, the model can fully learn spatiotemporal features and process multiple spatial directions, resulting in high-precision, high-efficiency, and robust change detection. In segmenting dynamic scenes (e.g., UAV-based survey), this structural improvement ensured trade-off between robust performance and computational efficiency image analysis tasks.

#### 2.5.2. Training of the Networks

All semantic segmentation models were trained using HR images processed in Section 2.3. All training was executed on a server equipped with an NVIDIA Tesla V100 GPU (32 GB memory), an Intel^®^ Xeon^®^ E5-2698 v4 processor, and Ubuntu 20.04 as the operating system. The implementation was based on the PyTorch 2.2.1 framework with CUDA 12.2 and cuDNN 8.8.0 libraries. Input images were in 256 × 256 pixels, and the models were optimized using mini-batches of size 8. The initial learning rate was set to ${\;1\times 10}^{-5}$, with training carried out for 20,000 iterations. Optimization was performed with the Adam algorithm in combination with a cross-entropy loss function. To accelerate convergence and enhance accuracy, several encoder–decoder architectures (e.g., UNet++, DPT, and ChangeMamba) were initialized with publicly available pretrained weights.

#### 2.5.3. Evaluation Metrics

Pixel-level semantic segmentation of tobacco maturity enabled fine-grained characterization of agronomic traits, including leaf color distribution and lesion area quantification, critical for assessing physiological states and harvest readiness. To evaluate segmentation accuracy, IoU metric was employed, which commonly used to quantify alignment between predicted segmentation masks and ground-truth annotations, addressing both class-specific and spatial agreement.

(7) $$IoU=\frac{TP}{TP+FN+FP}$$

where TP (true positives), FP (false positives), and FN (false negatives) denoted pixel-wise classification outcomes. This study calculated both IoU and mean IoU (mIoU) to comprehensively evaluate the image background, the ripe part of the tobacco leaf, and the unripe part of the image.

## 3. Result

### 3.1. Dataset and Experiment Setting

#### 3.1.1. Dataset Description

The dataset collected with drone comprises 840 HR images (256 × 256 pixels), acquired directly from tobacco plantations under varying illumination and growth conditions. The HR dataset was divided into 735 images for training and 105 images for testing. data augmentation techniques such as 180° rotation, vertical and horizontal flipping, mirroring, and scaling by ratios of 0.6, 0.7, 0.8, and 0.9 were applied [48]. These operations substantially increased dataset diversity, thereby improving the diversity of the dataset so that enhancing the performance and robustness of model. Specifically, after augmentation, the dataset size increased by a factor of 30, resulting in 22,050 training images and 3150 test images.

To simulate real-world imaging constraints such as motion blur from field equipment or resolution limitations of agricultural sensors, corresponding LR images were synthesized through controlled degradation of HR images. The degradation process was modeled as [49]:

(8) $$g=(f⊗h){↓}_{s}^{bicubic}+\eta$$

In this context, g and f denote the LR image and HR counterpart, respectively. The function h represents the point spread function under uniform linear motion to characterize the degradation effect. The operator ⊗ indicates convolution process. The downsampling step, symbolized by ↓ and s represents scaling factor. Bicubic interpolation is applied for upscaling, while η denotes Gaussian white noise introduced to simulate acquisition-related randomness.

To enhance training efficiency, *l*sub × *l*sub patches were cropped from LR images, paired with corresponding HR patches of size *sl*sub × *sl*sub, where s represented the magnification factor. These paired sub-images served as training samples, optimizing the capacity of model to generalize by capturing diverse spatial features and scaling variations across the dataset. After processing process, the dataset was split into 70% training, 10% validation, and 10% test sets.

#### 3.1.2. Experimental Setup

A core objective was to improve the visual quality of LR images for subsequent segmentation tasks. Five deep learning-based SRR algorithms—SRCNN, SRFBN, EDSR, RDN and RCAN—were evaluated. Each algorithm employed distinct network designs to learn a nonlinear mapping from LR to HR images. The LR–HR image pairs were used to train each SRR model, with HR images serving as ground-truth references. All SRR outputs were assessed using PSNR and SSIM. PSNR quantified pixel-level reconstruction accuracy relative to the original HR image, whereas SSIM evaluates structural consistency. Models with higher PSNR and SSIM values indicated superior reconstruction performance.

After generating super-resolved images, an advanced tobacco segmentation model was utilized to differentiate tobacco leaves from surrounding weeds and background. eight state-of-the-art semantic segmentation architectures were initially considered. The final chosen model incorporated specialized modules to handle the visual complexity of tobacco fields, where leaves and weeds often shared similar color and texture.

All SRR experiments were conducted in a Python 3.10 environment, leveraging the PyTorch deep learning framework. An NVIDIA RTX 4090 GPU with 64 GB of dedicated memory was utilized to accelerate training for both SRR and segmentation models.

### 3.2. Analysis of the Super-Resolution Reconstruction

The results of the SRR experiments are summarized in Table 1, which presents the PSNR and SSIM values for six methods under a magnification factor of 4. Bicubic interpolation served as the baseline, achieving a PSNR of 23.90 dB and an SSIM of 63.44%. In contrast, SRCNN achieved a noticeable performance improvement due to its ability to learn end-to-end mappings between LR and HR images through a straightforward three-layer architecture. This structure was particularly effective for tobacco field images, where clear leaf edges and smooth texture regions benefit from localized convolutional learning. Similar studies in crop segmentation have also reported that SRCNN outperforms traditional interpolation methods when dealing with repetitive agricultural patterns and moderate image complexity. Notably, SRFBN relying on a feedback mechanism for iterative refinement enhances the PSNR by 24.89 dB and SSIM by 68.95% compared to SRCNN. Moving toward the top-performing models, EDSR and RDN show further improvements, aided by residual connections and dense feature aggregation. These architectural elements allowed the networks to retain and reuse salient feature maps, leading to more accurate reconstructions of edges and textures. The use of residual dense blocks that allowed the RDN to extract and fuse multi-level features across layers demonstrated consistently high metrics. This deep feature enhanced the ability to reconstruct fine textures and structural details of leaf veins and boundaries vary in scale in tobacco images while also reducing common artifacts like noise amplification and over-smoothing. Ultimately, RCAN, which employed a channel attention mechanism within residual blocks, achieved the highest PSNR of 24.98 dB and the highest SSIM of 69.48%. This indicated an enhanced ability to recover both fine details and broader structural information from LR inputs. The channel attention module specifically tailored the network’s focus on critical features, facilitating more precise reconstruction of challenging textures and subtle color gradations.

**Figure 3:**
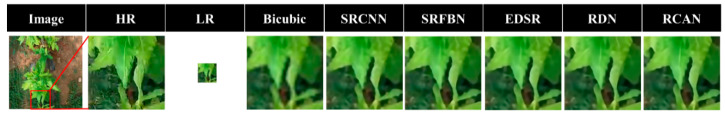
Comparison of the visual effects of the reconstructed images based on the six methods. The first column shows the original HR image with a selected region of interest and its magnified patch. While the visual differences are subtle, quantitative image quality metrics in Table 1 and downstream segmentation results in Table 2 demonstrate consistent improvements.

Figure 3 provides a side-by-side visualization of the reconstructed images, highlighting variations in edge clarity, color fidelity, and texture restoration among different SRR methods. Bicubic produced noticeably blurred edges, underscoring its limitations in capturing fine-grained features. In contrast, SRCNN and SRFBN recovered sharper contours but exhibited slight artifacts in uniform regions. EDSR and RDN revealed well-preserved textures, effectively reducing noise while retaining leaf boundaries. RCAN, which integrated channel attention mechanisms, offered the most visually convincing reconstruction, with consistently crisp edges and accurate color transitions that closely approximated the original high-resolution reference. The visual effect and the quantitative results were consistent to a certain extent, which showed the rationality of PSNR and SSIM as evaluation indicators of this study.

**Table 2:** Segmentation results of different SRR-generated testsets by using ensemble model.

Metrics (%)	HR	Bicubic	SRCNN	SRFBN	EDSR	RDN	RCAN
mIoU	90.75	82.79	86.46	87.86	88.27	88.96	89.18
IoU Green	94.90	88.25	91.68	92.27	93.04	93.23	93.44

### 3.3. Analysis of Semantic Segmentation

#### 3.3.1. Single Model Performance

Table 3 provides a comprehensive overview of segmentation outcomes for various decoder–encoder architectures, evaluated in terms of IoU for three classes green (background), white (other vegetation or weeds), and brown (tobacco leaves) and the mIoU. A higher IoU indicated better segmentation accuracy. The results were grouped by encoder category: CNN-based, Transformer-based, and Mamba-based.

The CNN-based encoders exhibited strong performance when paired with deeper architectures, exemplified by UNet++ with ResNeXt101_32x8d, which yielded an mIoU of 87.97%. Sophisticated skip connections and extended feature extraction pipelines in this architectures can preserve intricate spatial details, resulting higher IoU values. Transformer-based encoders significantly raised segmentation accuracy. Notably, DPT combined with DINOv2(vit_b) achieved an mIoU of 90.18%, underscoring how global context modeling, inherent in Transformer architectures, enhanced the delineation of subtle color gradients and complex shapes. Mamba-based encoders likewise performed competitively, with ChangeMamba and UperNet frameworks reaching mIoUs close to 90%. Their state-space formulations allowed the model to effectively aggregate features across multiple scales. Although slightly lower than the best Transformer results, Mamba-based methods still demonstrated robust discrimination of tobacco leaves from varied backgrounds.

#### 3.3.2. Ensemble Learning Approach

The Ensemble Segmentation Model that integrated 27 segmentation models, including CNN, Transformer, and Mamba architectures, improved the accuracy and robustness of tobacco segmentation. By combining multiple model predictions, this ensemble approach effectively captured complementary spatial, structural, and contextual features, reducing errors and variability typically associated with individual models. This led to more precise, reliable segmentation outcomes, especially in complex and challenging scenarios common in tobacco plant image analysis [50].

For ensemble fusion specifics, the framework predefined 27 different encoder–decoder segmentation models and selected the optimal ones based on validation set performance thresholds. As shown in Table 3, the final ensemble model consists of 17 + 19 + 20 + 22, corresponding to UNet + SegFormer(mit_b5), DPT + DINOv2(vit_1), DPT + DINOv2(vit_b), and ChangeMamba + VMamba(base). The ensemble approach supports probability averaging or majority voting, and the default setting with uniform model weights was applied in this study. Horizontal and vertical flip TTA was used during inference. No additional calibration (e.g., temperature scaling or bias correction) was applied, as preliminary experiments showed negligible differences. According to Table 3, each model individually attained mIoU values near or above 89%, indicating strong segmentation potential. Specifically, UNet + SegFormer(mit_b5) exceled in capturing boundary details through its U-shaped decoder structure, while DPT + DINOv2(vit_1) refined global context to effectively differentiate subtle color variations between tobacco leaves and weeds. DPT + DINOv2(vit_b) further built on this Transformer-based approach by adopting a larger backbone (vit_b), enabling superior long-range modeling in scenes with overlapping foliage. ChangeMamba + VMamba (base) adopted a state-space formulation to capture complex multi-scale spatial dependencies, which helped reduce misclassification in patches where leaf edges and background appear visually similar. This synergistic approach elevated the model’s capacity to separate similarly hued tobacco and weed areas, leading to an mIoU of 90.75%, surpassing the best single-model performance. The ensemble particularly exceled in delineating tobacco leaves with irregular edges or fine texture variations, as each contributing model added its own specialized perspective. Consequently, the integrated method achieved more stable and accurate segmentation, underscoring the value of ensemble learning for real-world applications where color and texture overlaps present notable classification challenges.

Based on the mIoU and IoU Green metrics discussed in this study, segmentation performance of the ensemble model across multiple test sets: the HR baseline, a Bicubic-based set, and five SR test sets reconstructed by SRCNN, SRFBN, EDSR, RDN, and RCAN. As depicted in Table 2, the HR test set achieves the best overall segmentation, yielding an mIoU of 90.75% and IoU Green of 94.90%. By contrast, the Bicubic test set shows notably lower scores, with an mIoU of 82.79%, reflecting the adverse effect of simple interpolation on pixel-wise classification. Among the SRR-based test sets, RCAN delivers the highest mIoU at 89.18%, followed closely by RDN at 88.96%. Both methods also perform well in IoU Green 93.44% and 93.23%, respectively, indicating that they effectively restore fine detail in tobacco leaves compared to simpler networks like SRCNN or SRFBN. Overall, the advanced SRR approaches (RDN, RCAN) substantially narrow the performance gap relative to the HR baseline, underscoring their capability to enhance image quality for segmentation purposes.

**Figure 4:**
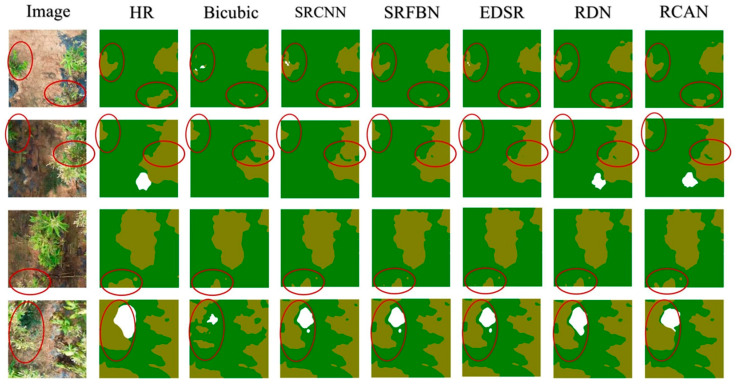
Qualitative comparison of segmentation results across various test sets. (The red circles indicate regions containing tobacco leaves. While certain methods, such as Bicubic fail to fully detect the target objects within these circled areas, others like RDN and RCAN show better segmentation performance, correctly identifying the objects in most cases).

Figure 4 provides a visual comparison of the segmentation maps under different SR reconstruction techniques. The HR images align most closely with the ground truth, accurately delineating leaf boundaries without significant omissions. Bicubic, on the other hand, exhibits conspicuous artifacts and blurred edges, resulting in partial misclassification of tobacco leaves, particularly near complex or overlapping foliage (red circled areas). By contrast, the RDN and RCAN outputs recover crisper edges and leaf contours, which the ensemble model can segment more reliably. White-colored artifacts in Bicubic’s masks are visibly reduced in the SRR-based sets, confirming that higher-quality reconstructed images allow the model to preserve subtle leaf textures.

**Figure 5:**
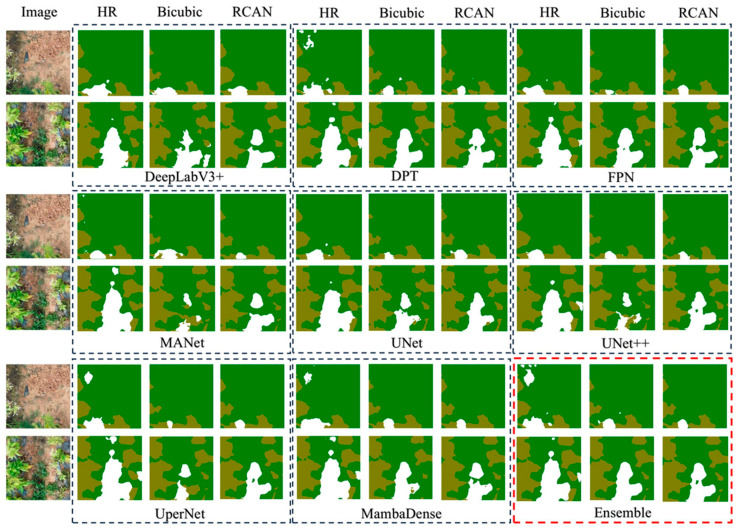
Segmentation prediction of different segmentation networks on three test set: HR, Bicubic, and SR. (The brown regions represent segmented tobacco leaves, while the white regions indicate weed. The results for each network are compared to the HR baseline, which serves as a reference for accurate segmentation).

Figure 5 shows a comparative overview of segmentation results across multiple architectures—DeepLabV3+, DPT, FPN, MANet, UNet, UNet++, UperNet, MambaDense, and an ensemble approach—applied to three test sets: the HR images, Bicubic generated images, and SR images generated by RCAN. A clear visual distinction emerges when comparing Bicubic-based results with the HR and RCAN columns. The Bicubic outputs often exhibit blurred boundaries around leaf edges, causing partial misclassifications or omissions of tobacco plants. This effect is most evident in networks like DeepLabV3+ or MANet, where leaf contours in the Bicubic column appear fragmented or fused with surrounding vegetation. By contrast, the RCAN column shows sharper contours and reduced artifacting, enabling these same networks to more accurately delineate leaf boundaries. Among the segmentation architectures, UNet++ and DPT are particularly adept at capturing intricate spatial details, evidenced by their clearer separation of leaves and background in the RCAN column. Similarly, MambaDense preserves fine-grained textures in high-density foliage areas, demonstrating robust performance even when vegetation and leaf color intensities overlap. In the final Ensemble column (outlined in red), multiple architectures are fused to exploit their complementary strengths in both global context modeling and local boundary precision. The ensemble maps are consistently the most aligned with the HR reference, indicating fewer misclassifications and better overall coverage of tobacco leaves.

## 4. Discussion

### 4.1. Impact of Magnification Factor

The performance of SRR is closely related to the magnification factor, which determines the scaling ratio from LR to HR. Variations in this factor can significantly influence an ability of model to reconstruct fine image details, which is critical for downstream tasks such as tobacco leaf segmentation. Previous studies [51] have demonstrated that magnification levels have a substantial impact on both visual quality and segmentation metrics, including the mIoU.

To investigate this relationship, a series of experiments were conducted to evaluate how different magnification factors affect segmentation accuracy. HR tobacco images with dimensions of 256 × 256 were initially downsampled to produce an LR set (x1-LR) at a resolution of 64 × 64, simulating realistic close-range capturing conditions. SRR models were subsequently trained on this LR set using magnification factors ranging from 2 to 5, generating SR sets with resolutions of x2-SR (128 × 128), x3-SR (192 × 192), x4-SR (256 × 256), and x5-SR (320 × 320), respectively.

**Figure 6:**
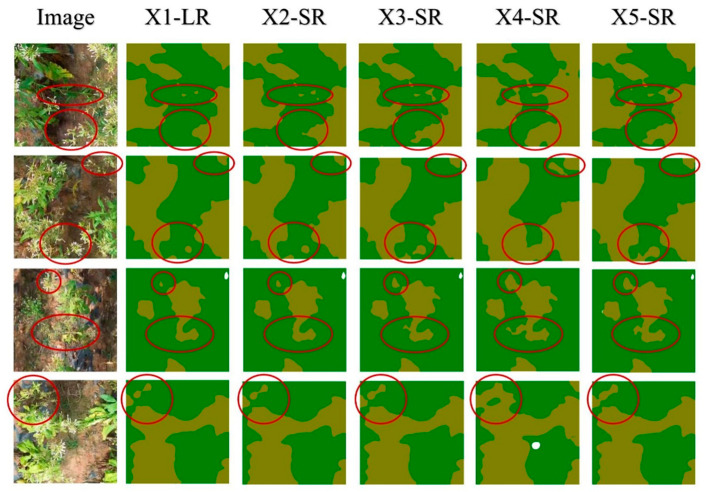
Segmentation results of images with different magnification factors. (The brown regions indicate the tobacco field. Red circles are used to compare the reconstructed details at different magnification factors).

**Figure 7:**
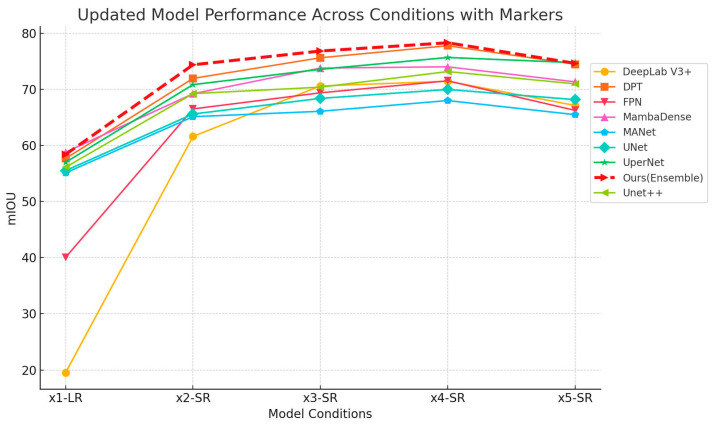
Evaluating the accuracy of tobacco leaves segmentation across different magnifications.

Segmentation performance was assessed using an ensemble segmentation model trained on the original HR tobacco leaf dataset. Figure 6 illustrates segmentation results across different SR magnifications, showing progressively refined segmentation boundaries as the magnification factor increases. Additionally, Figure 7 quantitatively presents the segmentation accuracy (mIoU) across varying magnification conditions, clearly indicating performance improvements up to a 3x magnification factor, after which improvements plateau. Notably, the proposed ensemble segmentation method consistently achieved the highest mIoU values across all magnification levels, closely followed by DPT and MambaDense. At lower magnifications (2×), DPT slightly outperformed MambaDense, maintaining marginally better segmentation accuracy at higher magnifications. DeepLab V3+ demonstrated comparatively lower accuracy, consistently trailing other algorithms significantly. Despite superior performance at higher magnifications, computational demands also increased substantially. Thus, for practical tobacco leaf segmentation, a 4× magnification factor appears optimal, balancing segmentation precision with computational efficiency. It is significant for real-world UAV-based agricultural monitoring, as it ensures high segmentation accuracy without incurring excessive processing time or hardware demands, making the approach feasible for large-scale, time-sensitive field applications.

### 4.2. Computational Efficiency and Sensor Constraints

Beyond reconstruction accuracy, computational efficiency is a critical consideration for the deployment of SRR models and segmentation models in agricultural practice. For SRR models, inference time varies substantially across architectures: for a 256 × 256 input, SRCNN and EDSR require approximately 1.5 ms, SRFBN 2 ms, RDN 5 ms, while RCAN exceeds 8 ms per patch. Lightweight models are faster but less accurate, whereas advanced architectures achieve superior fidelity at the cost of increased computational burden. For segmentation models, the inference speed of the ensemble segmentation model under this study’s experimental setting is approximately 2.5 images per second for 256 × 256 inputs. These trade-offs raise challenges for real-time or on-board UAV applications, where computational resources are limited [52]. To address these challenges, recent studies have investigated acceleration strategies such as model pruning, quantization, and lightweight backbones [53]. Several works also report near real-time performance on embedded GPUs (e.g., NVIDIA Jetson TX2), although these improvements often come at the cost of reduced accuracy [54]. Together, these advances indicate that although current high-fidelity models may be too computationally demanding for UAV deployment, emerging acceleration strategies provide promising avenues to adapt SRR–segmentation frameworks for field-deployable systems.

In addition to computational aspects, sensor-related constraints also limit UAV-based monitoring. Consumer-grade UAVs typically face restrictions in camera resolution, flight endurance, and sensitivity to environmental conditions such as wind or variable illumination. High-resolution sensors could alleviate some issues but increase cost, energy consumption, and data volume. Our results demonstrate that SRR offers a cost-effective alternative to compensate for such limitations. To further evaluate its applicability, we extended our analysis to UAV imagery collected at a higher flight altitude of 10 m, which yields lower-resolution inputs compared to the 3 m baseline dataset. These 10 m images were first enhanced using the trained RCAN model and subsequently evaluated with the same ensemble segmentation model. The segmentation accuracy improved, with mIoU rising from 75.12% at the original 10 m resolution to 79.85% after SRR enhancement. This result confirms that the proposed approach is effective not only for synthetically degraded low-resolution inputs but also for operational UAV imagery acquired under in situ field conditions. Overall, this finding underscores the potential of SRR to enhance UAV imagery acquired at higher altitudes, enabling accurate crop-weed discrimination without requiring expensive UAV platforms.

### 4.3. Impact of the Gaussian Blur

Different sizes of the Gaussian blur kernel significantly affect the performance of segmentation models by altering image clarity and noise levels. This study assessed tobacco leaf segmentation performance across various degrees of Gaussian blur, using kernel sizes of 11 (Blur_lv1), 15 (Blur_lv2), 21 (Blur_lv3), and 25 (Blur_lv4) to simulate realistic image degradation like motion blur and atmospheric disturbances commonly encountered in UAV-based agricultural monitoring.

**Figure 8:**
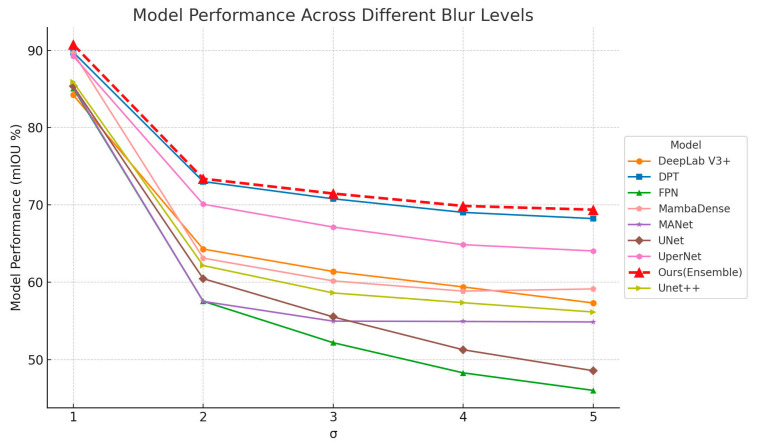
Evaluation of tobacco leaf segmentation accuracy under different Gaussian blur levels.

Figure 8 illustrates the relationship between Gaussian blur levels and segmentation accuracy, quantified by mIoU, indicating that accuracy consistently declined as the Gaussian blur kernel size increased. At the smallest blur level (Blur_lv1, kernel size 11), the Ensemble model achieved an mIoU of approximately 89%, reflecting high segmentation precision. However, as the blur kernel increased to Blur_lv2 (kernel size 15), a noticeable performance drop occurred, reducing the Ensemble model’s mIoU to approximately 70%. Further increases in blur kernel sizes to Blur_lv3 (kernel size 21) and Blur_lv4 (kernel size 25) led to additional but less drastic declines, stabilizing around 68% and 65%, respectively.

Among individual models, performance degradation varied significantly. Models such as MambaDense, DPT, and UperNet exhibited substantial performance drops, with mIoU reductions of roughly 25–30% from the initial blur level to the highest blur level tested. Despite these challenges, the Ensemble model consistently demonstrated superior robustness, outperforming all other segmentation models across all blur conditions by maintaining the smallest reduction in mIoU.

Although moderate Gaussian blur (Blur_lv1) effectively reduced noise and simplified boundary detection, excessive blurring at higher levels (Blur_lv3 and Blur_lv4) negatively impacted the models’ ability to distinguish critical edges and intricate textures inherent to tobacco leaves so that resulting in notable decreases in segmentation accuracy [55]. Therefore, in practical UAV-based applications, applying a light Gaussian blur with a kernel size around 11 (Blur_lv1) is recommended, as it provided an effective trade-off between noise suppression and preservation of essential image details required for accurate segmentation.

### 4.4. Impact of Gaussian Noise

In this experiment, different intensities of Gaussian noise were leveraged to evaluate their impacts on the segmentation outcome of SR tobacco leaf images. Gaussian noise intensity was varied through different values of standard deviation (σ), directly affecting both image clarity and segmentation accuracy. Five levels of noise intensity were examined, with σ values set at 1 (Noise_lv0), 2 (Noise_lv1), 3 (Noise_lv2), 4 (Noise_lv3), and 5 (Noise_lv4), representing progressively higher noise interference.

**Figure 9:**
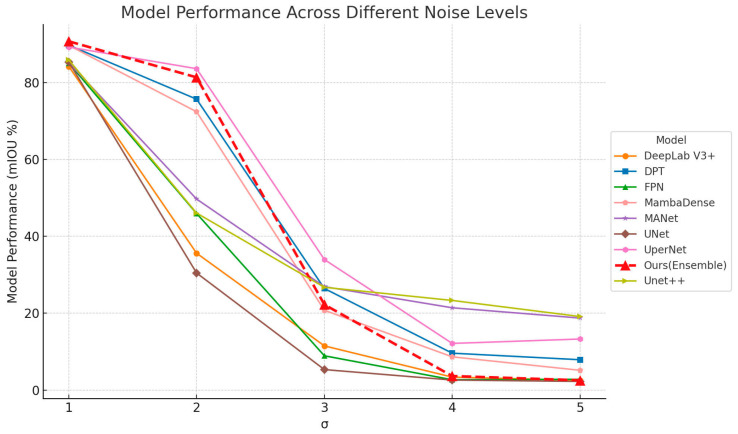
Evaluation of segmentation performance under varying levels of Gaussian noise.

As shown in Figure 9, when σ = 1, corresponding to the lowest noise level, the Ensemble model achieved the best segmentation accuracy across evaluation metric of mIoU of approximately 84%. Increasing the noise intensity to σ = 2 resulted in a noticeable but moderate drop in mIoU to about 55% for the Ensemble model. However, individual models such as DeepLab V3+, DPT, and MambaDense showed significantly larger decreases, with mIoU values dropping below 40%, indicating these models’ greater susceptibility to noise. Further increases in noise levels sharply degraded segmentation performance across all models. At σ = 3, segmentation accuracy drastically fell below 20% for most models, as the high-intensity Gaussian noise severely obscured essential visual cues such as leaf contours, textures, and color gradients—making it difficult for models to distinguish between tobacco and weed regions. Only the Ensemble and U-Net++ models maintained marginally better performance, likely due to their stronger feature extraction and multi-scale aggregation capabilities. The Ensemble model benefits from integrating diverse architectural strengths, while U-Net++’s nested skip connections help preserve spatial information, offering slightly greater resilience to noise-induced degradation. At σ values of 4 and 5, segmentation quality significantly deteriorated, resulting in mIoU values approaching 0% for most models. This pronounced reduction in accuracy highlights the detrimental effect of high-intensity Gaussian noise on image segmentation tasks. The escalating noise levels severely obscured critical edges, structural details, and intricate textures of tobacco leaves, making precise segmentation increasingly challenging. Quantitatively, the mIoU dropped from approximately 84% at σ = 1 to below 20% at σ = 3, and approached 0% at σ = 5, indicating a sharp degradation in model reliability under heavy noise interference. In practical UAV-based remote sensing applications, even moderate sensor noise or environmental interference such as low-light conditions, wind-induced motion or hardware limitations can significantly compromise segmentation performance. The findings highlight the urgent need for more noise-resilient models and image enhancement strategies, such as denoising pre-processing or robust training pipelines, to ensure accurate field-scale weed and crop discrimination under suboptimal imaging conditions.

## 5. Conclusions

Accurate weed detection in tobacco fields remains a major challenge due to LR UAV imagery and the visual similarity between tobacco plants and surrounding weeds. This study aims to improve segmentation precision by enhancing image clarity and model robustness under complex field conditions. An innovative approach combining deep learning-based SRR and semantic segmentation for accurately distinguishing tobacco plants from weeds is introduced, utilizing UAV remote sensing imagery. Initially, SRR techniques significantly improved image resolution from low-quality UAV-captured images, subsequently enabling the effective application of semantic segmentation models. Five distinct SRR models, including SRCNN, SRFBN, EDSR, RDN, and RCAN, were rigorously evaluated, with RCAN demonstrating superior performance in reconstructing fine details and achieving the highest PSNR and SSIM values. Using RCAN for image enhancement can theoretically improve segmentation accuracy by restoring finer structural features, potentially boosting classification performance by up to 7–8% compared to using raw or bicubic-interpolated images.

A comprehensive assessment of 27 semantic segmentation models, encompassing CNN-based, Transformer-based, and Mamba-based architectures, revealed the robust capabilities of Transformer models, notably DPT integrated with DINOv2, which achieved exceptional accuracy. Furthermore, an ensemble approach combining the best-performing models from each category resulted in significantly improved segmentation precision, effectively overcoming challenges related to visual similarity between tobacco plants and weeds, as well as environmental variability.

The impacts of magnification levels, Gaussian blur, and Gaussian noise on segmentation accuracy are also explored in this paper. The results highlighted the optimal magnification factor as 4x, balancing image clarity and computational efficiency, while emphasizing the detrimental effects of excessive blur and noise on segmentation performance. The ensemble segmentation model consistently demonstrated enhanced resilience across varying image conditions. Building upon these findings, the originality of this study lies in being the first to apply UAV-based SRR with semantic segmentation to tobacco fields, systematically evaluating newly emerging Transformer- and Mamba-based models, and open-sourcing an annotated dataset that supports reproducibility and benefits the broader research community.

Future directions will involve refining the integration of SRR and semantic segmentation, enhancing model performance across diverse agricultural scenarios and environmental conditions. Additionally, further research will extend pixel-level analysis to support precise yield estimation, potentially improving agricultural productivity, optimizing herbicide usage, and promoting environmental sustainability in tobacco cultivation.

## Figures and Tables

**Table 1:** Evaluation metrics of different methods on the LR testsets with the magnification factor of 4.

Metrics	Bicubic	SRCNN	SRFBN	EDSR	RDN	RCAN
PSNR (dB)	23.90	24.61	24.89	24.96	24.97	24.98
SSIM (%)	63.44	67.81	68.95	69.36	69.47	69.48

**Table 3:** Segmentation performance of various models.

#	Decoder	Encoder	IoU Green	IoU White	IoU Brown	mIoU
CNN-based Encoder						
1	FPN	EfficientNet-b5	75.18	63.73	78.46	85.09
2	UNet	EfficientNet-b5	76.39	64.52	79.17	85.36
3	DeepLabV3+	EfficientNet-b5	71.04	59.04	75.34	84.21
4	Unet++	EfficientNet-b5	77.49	64.91	79.69	85.88
5	FPN	ResNeXt101_32x8d	77.95	66.06	80.26	86.65
6	UNet	ResNeXt101_32x8d	78.64	66.16	80.53	86.37
7	DeepLabV3+	ResNeXt101_32x8d	75.96	64.38	78.95	86.78
8	UNet++	ResNeXt101_32x8d	79.93	67.70	81.54	87.97
9	FPN	ResNet101	75.32	63.30	78.36	86.11
10	UNet	ResNet101	77.34	64.66	79.54	86.33
11	DeepLabV3+	ResNet101	74.35	60.94	77.08	86.59
12	UNet++	ResNet101	78.82	66.39	80.64	86.64
13	MANet	EfficientNet-b5	73.82	63.98	78.08	85.23
14	MANet	ResNeXt101_32x8d	77.71	66.20	80.24	86.40
15	MANet	ResNet101	76.21	64.48	79.12	86.09
Transformer-based Encoder						
16	FPN	SegFormer(mit_b5)	77.47	64.00	79.33	89.34
17	UNet	SegFormer(mit_b5)	75.49	64.50	78.88	89.41
18	MANet	SegFormer(mit_b5)	77.52	64.25	79.44	88.91
19	DPT	DINOv2(vit_1)	80.67	67.99	81.87	90.04
20	DPT	DINOv2(vit_b)	80.50	66.79	81.37	90.18
21	DPT	DINOv2(vit_s)	80.10	66.87	81.31	89.75
Mamba-based Encoder						
22	ChangeMamba	VMamba (base)	78.27	65.73	80.25	89.69
23	ChangeMamba	VMamba (tiny)	78.38	66.23	80.48	89.28
24	ChangeMamba	VMamba (small)	78.09	65.17	79.98	89.49
25	UperNet	VMamba (base)	77.92	64.38	79.61	89.26
26	UperNet	VMamba (tiny)	78.17	65.31	80.06	89.28
27	UperNet	VMamba (small)	77.53	64.33	79.47	89.52
Ensemble Model						
17 + 19 + 20 + 22 (ours)			94.90	81.43	95.91	90.75

## Data Availability

Data will be made available on request.

## References

[1] Ben Jebli M., Boussaidi R. (2024). Empirical evidence of emissions discourse related to food, beverage, and tobacco production in leading manufacturing nations. Environ. Sci. Pollut. Res..

[2] Zafeiridou M., Hopkinson N.S., Voulvoulis N. (2018). Cigarette smoking: An assessment of tobacco’s global environmental footprint across its entire supply chain. Environ. Sci. Technol..

[3] Hendlin Y.H., Bialous S.A. (2020). The environmental externalities of tobacco manufacturing: A review of tobacco industry reporting. Ambio.

[4] Guzel M., Turan B., Kadioglu I., Basturk A., Sin B., Sadeghpour A. (2024). Deep learning for image-based detection of weeds from emergence to maturity in wheat fields. Smart Agric. Technol..

[5] Rezaei E.E., Webber H., Asseng S., Boote K., Durand J.L., Ewert F., Martre P., MacCarthy D.S. (2023). Climate change impacts on crop yields. Nat. Rev. Earth Environ..

[6] Lencucha R., Drope J., Magati P., Sahadewo G.A. (2022). Tobacco farming: Overcoming an understated impediment to comprehensive tobacco control. Tob. Control.

[7] Sunil G.C., Upadhyay A., Zhang Y., Howatt K., Peters T., Ostlie M., Aderholdt W., Sun X. (2024). Field-based multispecies weed and crop detection using ground robots and advanced YOLO models: A data and model-centric approach. Smart Agric. Technol..

[8] Gupta S.K., Yadav S.K., Soni S.K., Shanker U., Singh P.K. (2023). Multiclass weed identification using semantic segmentation: An automated approach for precision agriculture. Ecol. Inform..

[9] Lecours N. (2014). The harsh realities of tobacco farming: A review of socioeconomic, health and environmental impacts. Tobacco Control and Tobacco Farming: Separating Myth from Reality.

[10] Sharma A.K., Sharma M., Sharma A.K., Sharma M. (2023). Mapping the impact of environmental pollutants on human health and environment: A systematic review and meta-analysis. J. Geochem. Explor..

[11] Lins H.A., Souza M.d.F., Batista L.P., Rodrigues L.L.L.d.S., da Silva F.D., Fernandes B.C.C., de Melo S.B., das Chagas P.S.F., Silva D.V. (2024). Artificial intelligence for herbicide recommendation: Case study for the use of clomazone in Brazilian soils. Smart Agric. Technol..

[12] Coulibaly S., Kamsu-Foguem B., Kamissoko D., Traore D. (2022). Deep learning for precision agriculture: A bibliometric analysis. Intell. Syst. Appl..

[13] Lu Y., Yang W., Zhang Y., Chen Z., Chen J., Xuan Q., Wang Z., Yang X. Understanding the Dynamics of DNNs Using Graph Modularity. Proceedings of the European Conference on Computer Vision (ECCV).

[14] Huang J., Ma Z., Wu Y., Bao Y., Wang Y., Su Z., Guo L. (2025). YOLOv8-DDS: A lightweight model based on pruning and distillation for early detection of root mold in barley seedling. Inf. Process. Agric..

[15] Wu Y., Huang J., Wang S., Bao Y., Wang Y., Song J., Liu W. (2025). Lightweight Pepper Disease Detection Based on Improved YOLOv8n. AgriEngineering.

[16] Guo L., Huang J., Wu Y. (2025). Detecting rice diseases using improved lightweight YOLOv8n. Trans. Chin. Soc. Agric. Eng..

[17] Zheng Y.Y., Kong J.L., Jin X.B., Wang X.Y., Su T.L., Zuo M. (2019). CropDeep: The crop vision dataset for deep-learning-based classification and detection in precision agriculture. Sensors.

[18] Zhao F., He Y., Song J., Wang J., Xi D., Shao X., Wu Q., Liu Y., Chen Y., Zhang G. (2025). Smart UAV-assisted blueberry maturity monitoring with Mamba-based computer vision. Precis. Agric..

[19] Patel D., Gandhi M., Shankaranarayanan H., Darji A.D. (2022). Design of an Autonomous Agriculture Robot for Real-Time Weed Detection Using CNN. Advances in VLSI and Embedded Systems: Select Proceedings of AVES 2021.

[20] Babu V.S., Ram N.V. (2022). Deep residual CNN with contrast limited adaptive histogram equalization for weed detection in soybean crops. Trait. Du Signal.

[21] Gao J., Liao W., Nuyttens D., Lootens P., Alexandersson E., Pieters J. (2022). Transferring learned patterns from ground-based field imagery to predict UAV-based imagery for crop and weed semantic segmentation in precision crop farming. arXiv.

[22] Moazzam S.I., Khan U.S., Qureshi W.S., Nawaz T., Kunwar F. (2023). Towards automated weed detection through two-stage semantic segmentation of tobacco and weed pixels in aerial imagery. Smart Agric. Technol..

[23] Tufail M., Iqbal J., Tiwana M.I., Alam M.S., Khan Z.A., Khan M.T. (2021). Identification of tobacco crop based on machine learning for a precision agricultural sprayer. IEEE Access.

[24] Huang L., Wu X., Peng Q., Yu X. (2021). Depth semantic segmentation of tobacco planting areas from unmanned aerial vehicle remote sensing images in plateau mountains. J. Spectrosc..

[25] Xu B., Fan J., Chao J., Arsenijevic N., Werle R., Zhang Z. (2023). Instance segmentation method for weed detection using UAV imagery in soybean fields. Comput. Electron. Agric..

[26] Huang Y., Wen X., Gao Y., Zhang Y., Lin G. (2023). Tree Species Classification in UAV Remote Sensing Images Based on Super-Resolution Reconstruction and Deep Learning. Remote Sens..

[27] Zeng S., Qi D., Chang X., Xiong F., Xie S., Wu X., Liang S., Xu M., Wei X. (2025). Janusvln: Decoupling semantics and spatiality with dual implicit memory for vision-language navigation. arXiv.

[28] Liu Z., Han J., Liu J., Li Z.C., Zhai G. (2024). Neighborhood evaluator for efficient super-resolution reconstruction of 2D medical images. Comput. Biol. Med..

[29] Chi J., Wei X., Sun Z., Yang Y., Yang B. (2024). Low-Dose CT Image Super-resolution Network with Noise Inhibition Based on Feedback Feature Distillation Mechanism. J. Imaging Inform. Med..

[30] Chen H., Song J., Han C., Xia J., Yokoya N. (2024). Changemamba: Remote sensing change detection with spatio-temporal state space model. IEEE Trans. Geosci. Remote Sens..

[31] Zeng S., Chang X., Xie M., Liu X., Bai Y., Pan Z., Xu M., Wei X. (2025). FutureSightDrive: Thinking Visually with Spatio-Temporal CoT for Autonomous Driving. arXiv.

[32] Arun P.V., Herrmann I., Budhiraju K.M., Karnieli A. (2019). Convolutional network architectures for super-resolution/sub-pixel mapping of drone-derived images. Pattern Recognit..

[33] Aslahishahri M., Stanley K.G., Duddu H., Shirtliffe S., Vail S., Stavness I. (2021). Spatial super-resolution of real-world aerial images for image-based plant phenotyping. Remote Sens..

[34] Nogueira E.A., Felix J.P., Fonseca A.U., Vieira G. (2023). Deep Learning for Super Resolution of Sugarcane Crop Line Imagery from Unmanned Aerial Vehicles. International Symposium on Visual Computing.

[35] Palan V.A., Thakur S., Sumith N. (2025). Leveraging super-resolution technology in drone imagery for advanced plant disease diagnosis and prognosis. IEEE Access.

[36] Zhao F., Huang J., Liu Y., Wang J., Chen Y., Shao X., Ma B., Xi D., Zhang M., Tu Z. A deep learning approach combining super-resolution and segmentation to identify weed and tobacco in UAV imagery. Proceedings of the 2024 IEEE International Conference on Computer Science and Blockchain (CCSB).

[37] Li M., Zhong B., Ma K.K. (2022). MA-NET: Multi-scale attention-aware network for optical flow estimation. Proceedings of the ICASSP 2022—2022 IEEE International Conference on Acoustics, Speech and Signal Processing (ICASSP).

[38] Dong C., Loy C.C., He K., Tang X. (2015). Image super-resolution using deep convolutional networks. IEEE Trans. Pattern Anal. Mach. Intell..

[39] Lim B., Son S., Kim H., Nah S., Mu Lee K. Enhanced deep residual networks for single image super-resolution. Proceedings of the IEEE Conference on Computer Vision and Pattern Recognition Workshops.

[40] Zhang Y., Tian Y., Kong Y., Zhong B., Fu Y. Residual dense network for image super-resolution. Proceedings of the IEEE Conference on Computer Vision and Pattern Recognition.

[41] Sara U., Akter M., Uddin M.S. (2019). Image quality assessment through FSIM, SSIM, MSE and PSNR—A comparative study. J. Comput. Commun..

[42] Lin T.Y., Dollár P., Girshick R., He K., Hariharan B., Belongie S. Feature pyramid networks for object detection. Proceedings of the IEEE Conference on Computer Vision and Pattern Recognition.

[43] Xiao T., Liu Y., Zhou B., Jiang Y., Sun J. Unified perceptual parsing for scene understanding. Proceedings of the European Conference on Computer Vision (ECCV).

[44] Ronneberger O., Fischer P., Brox T. (2015). U-net: Convolutional networks for biomedical image segmentation. Medical Image Computing and Computer-Assisted Intervention–MICCAI 2015: 18th International Conference, Munich, Germany, 5–9 October 2015, Proceedings, Part III 18.

[45] Zhou Z., Rahman Siddiquee M.M., Tajbakhsh N., Liang J. (2018). Unet++: A nested u-net architecture for medical image segmentation. Deep Learning in Medical Image Analysis and Multimodal Learning for Clinical Decision Support: 4th International Workshop, DLMIA 2018, and 8th International Workshop, ML-CDS 2018, Held in Conjunction with MICCAI 2018, Granada, Spain, 20 September 2018, Proceedings 4.

[46] Chen L.C., Zhu Y., Papandreou G., Schroff F., Adam H. Encoder-decoder with atrous separable convolution for semantic image segmentation. Proceedings of the European Conference on Computer Vision (ECCV).

[47] Ranftl R., Bochkovskiy A., Koltun V. Vision transformers for dense prediction. Proceedings of the IEEE/CVF International Conference on Computer Vision.

[48] Goceri E. (2023). Medical image data augmentation: Techniques, comparisons and interpretations. Artif. Intell. Rev..

[49] Zhang K., Liang J., Van Gool L., Timofte R. Designing a practical degradation model for deep blind image super-resolution. Proceedings of the IEEE/CVF International Conference on Computer Vision.

[50] Zhang M., Chen T.E., Gu X., Kuai Y., Wang C., Chen D., Zhao C. (2023). UAV-borne hyperspectral estimation of nitrogen content in tobacco leaves based on ensemble learning methods. Comput. Electron. Agric..

[51] Xiang C., Wang W., Deng L., Shi P., Kong X. (2022). Crack detection algorithm for concrete structures based on super-resolution reconstruction and segmentation network. Autom. Constr..

[52] González D., Patricio M.A., Berlanga A., Molina J.M. (2022). A super-resolution enhancement of UAV images based on a convolutional neural network for mobile devices. Pers. Ubiquitous Comput..

[53] Jiang X., Wang N., Xin J., Xia X., Yang X., Gao X. (2021). Learning lightweight super-resolution networks with weight pruning. Neural Netw..

[54] Donapati R.R., Cheruku R., Kodali P. (2023). Real-Time Seed Detection and Germination Analysis in Precision Agriculture: A Fusion Model With U-Net and CNN on Jetson Nano. IEEE Trans. AgriFood Electron..

[55] Wang X., Liang X., Zheng J., Zhou H. (2019). Fast detection and segmentation of partial image blur based on discrete Walsh–Hadamard transform. Signal Process. Image Commun..

